# Recurrent labial adhesion and vaginal destruction treatment with ileal vaginoplasty and labia major flap: A rare case report

**DOI:** 10.1016/j.eucr.2023.102405

**Published:** 2023-04-21

**Authors:** Narjes saberi, Mohammadhosein Izadpanahi, Hossein Bahrami Samani

**Affiliations:** a Department of Urology, Isfahan Kidney disease Research Center, Isfahan University of Medical Sciences, Chaharbag Khjoo Street, 23 st,Shahid Ghasem Izadi Alley, Isfahan, Iran; b School of medicine, Al-zahraHospital, Isfahan University of Medical Sciences, Iran; c School of medicine, Isfahan University of Medical Science, Iran

**Keywords:** Labial adhesion, Ileal vaginoplasty, Flap, Recurrent

## Abstract

Labial adhesion (LA) and vaginal destruction are very rare conditions in women. We report a 40-year-old woman with severe LA and distal vaginal stenosis after a radical hysterectomy at the age of 35. Completely destruction of vaginal epithelium, severe recurrent LA, voiding symptoms, and chronic pelvic pain occurs for her due to the repeated vaginal dilatation and low estrogen level. A combination of ileal vaginoplasty (IV) and labia majora flap was used in two surgical stages for treatment. After surgery, the patient's urinary symptoms and pelvic pain were relieved and she was able to have sex with her partner.

## 1 Introduction

Labial adhesion is very rare in women of reproductive age. It is usually seen in children and menopausal women following a decrease in estrogen levels.^1^ It can be accompanied by adhesion and tightness of the distal vagina. Vaginal dilatation is usually used to treat severe and recurring strictures.^2^ To treat severe and recurrent cases that lead to the destruction of the labia minora and vaginal epithelium, more advanced surgical procedures and sometimes plastic surgery techniques are needed.^3^^,^^4^

## 2 Case report

A 40-year-old woman was referred to our center with LA and vaginal stricture. She had a past surgical history of radical hysterectomy and bilateral **oophorectomy** at the age of 35 for cervical cancer. The first time, one year after the hysterectomy, the patient presented with a complaint of labia and distal vagina adhesion and underwent surgery and vaginal dilatation several times with the use of topical estrogen. After that, the patient was referred to our center due to voiding symptoms (frequency, incomplete emptying, straining, and Post-void dribbling) and severe perineal pain. In vaginal examination complete LA was evident and the anterior and posterior epithelium of the vagina was destroyed. Vaginal and inner labia minora mucosa was atrophic and fibrotic. The adhesion of the vagina was initially in the distal part of the vagina, but after 7 sessions of surgery and dilation of the vagina with metal dilators, almost most of the anterior and posterior epithelium of the vagina was destroyed iatrogenically, and could not be repaired and thus she was scheduled for ileal vaginoplasty.

**Fig. 1:**
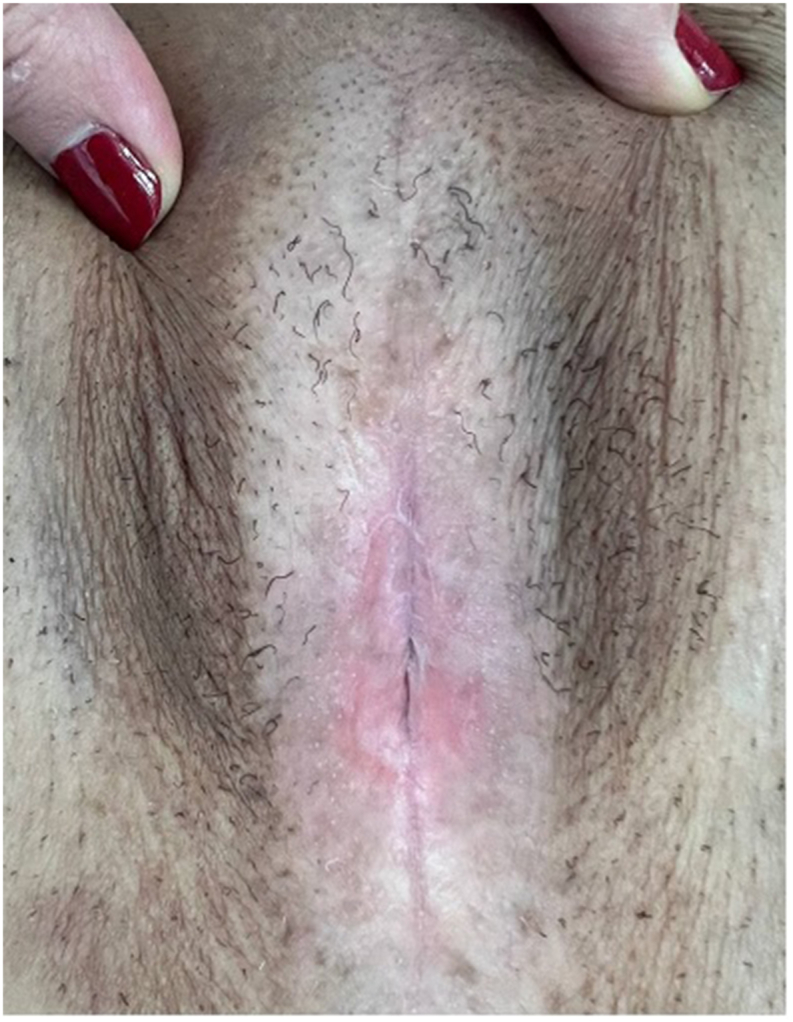
Labia and distal vagina adhesion.

**Fig. 2:**
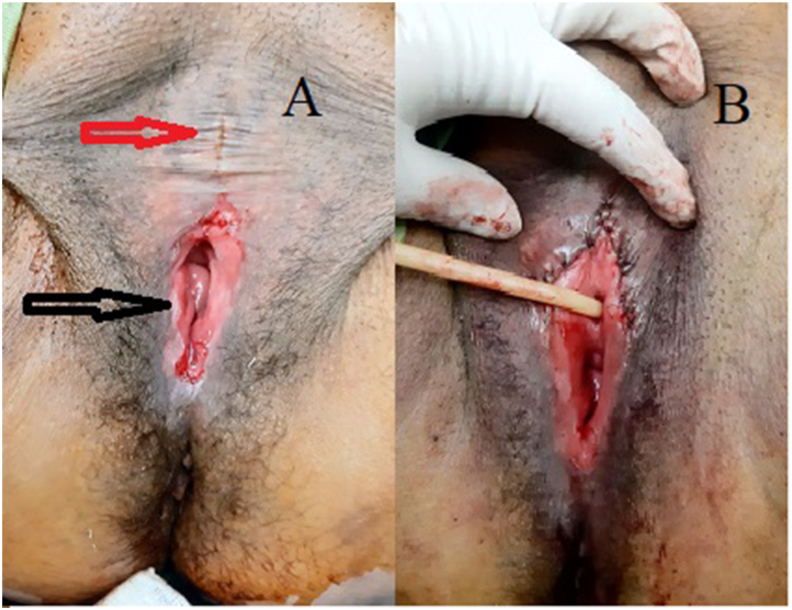
A: Red arrow: Adhesion of the labia minor around the urethra after the first surgery. Black arrow: Vaginal reconstruction with ileum. B: Using the labia majora flap for repair. (For interpretation of the references to colour in this figure legend, the reader is referred to the Web version of this article.)

The day before surgery she was admitted and received a complete intestinal prep. Under general anesthesia and in the lithotomy position, the anterior and posterior walls of the vagina were completely separated. Fibrous tissues and remnants of destroyed vaginal epithelium were removed. The vaginal cuff was opened. Then a midline longitudinal abdominal incision was made below the umbilicus and, 20 cm of the ileum was separated by preserving the terminal ileum and with a suitable vascular base. The intestinal anastomosis was performed and the separated ileum was brought from the vaginal cuff into the vaginal canal, and its edges were sutured to the perineum. The patient was discharged one week later without any complications with the prescription of reconstructed vaginal dilatation using a dilator and topical ointments. In the last follow-up one year after the operation, she was sexually active and the pelvic pains recovered, but she complained of voiding symptoms (incomplete emptying, straining, and Post-void dribbling). In the vaginal examination, there was a labia minor adhesion around the urethra, but the reconstructed vagina was completely open (Fig. 1). Due to the atrophic and fibrotic nature of the vaginal mucosa, the patient was a candidate for surgery and, the adhesions of the labia minor were released with a sharp dissection. After removing the atrophic and fibrotic vaginal mucosa and the remaining demolished labia minora, the labia majora was freed from the underlying tissues while maintaining the vascular base and was pulled inside and sutured to the healthy mucosa around the urethra (Fig. 2). In the last follow-up two months after surgery the patient was sexually active and had no voiding symptoms.

## 3 Discussion

Hysterectomy and bilateral **oophorectomy at lower age can lead to estrogen deficiency, LA, and distal vaginal stenosis.**^1^ Frequent and violent dilatation of the vagina and surgical separation of the labials can lead to the destruction of the vaginal epithelium and the perineum and *periurethral* mucosa. The primary treatment in cases of severe LA is to open the labia using surgery and the use of topical ointments.^5^ In this case, tissue samples were sent in both surgeries, and fibrosis and severe tissue inflammation were reported both times, but lichen sclerosis and other underlying skin diseases were not reported. It seems that the cause of the problem in the patient is bilateral **oophorectomy** at a young age and estrogen deficiency in its context. Of course, violent and repeated dilatations of the vagina with metal dilators can also be the cause of the patient's problem. There is a need for more advanced treatments and plastic surgery techniques in recurrent and refractory cases. In past studies, various flaps such as skin flaps from the perineum have been used.^3^ Vaginoplasty using a bowel segment is a safe and effective procedure that obtains excellent long-term results.^4^ One of the limitations of using the ileum for vaginal reconstruction is the impossibility of using it in people with a history of inflammatory bowel diseases and radiotherapy, and sometimes its mesial length is too short to pull it to the perineum which creates limitations. Another limitation is the small diameter of the ileum, which sometimes prevents sexual intercourse and there may be a need for frequent dilatation after the surgery.^4^ In our patient, IV was performed to treat adhesions and destruction of the vagina and to improve the patient's sexual status and the possibility of having sex. Due to the severe fibrosis and inappropriate tissue of the labia minora, the adhesions around the urethra recurred and we had to perform a second surgery and use a labia majora flap to correct it.
